# Nonlinear Wave Mixing Approach for Internal Damage Localization in Concrete

**DOI:** 10.3390/s26154856

**Published:** 2026-08-01

**Authors:** Klayne Dos Santos Silva, Vincent Garnier, Benoit Durville, Menes Badika, Sandrine Morin, David Marlot, Pierre Delvart, Ruben Edery, Cédric Payan

**Affiliations:** 1 French Authority for Nuclear Safety and Radiation Protection (ASNR), 92120 Montrouge, France; kkattiley@gmail.com (K.D.S.S.);; 2 Laboratory of Mechanics and Acoustics (LMA), UMR 7031, CNRS, Centrale Méditerranée, Aix-Marseille University, 13013 Marseille, France; 3 MISTRAS Group SAS, 94730 Sucy-en-Brie, France

**Keywords:** non-destructive testing, nonlinear acoustics, concrete, thermal damage, depth damage detection

## Abstract

Nonlinear acoustics has emerged as a sensitive and promising non-destructive approach to damage detection in concrete structures. This study proposes a method based on nonlinear acoustics that uses collinear mixing ultrasonic waves to detect and locate defects (microcracks, macrocracks, spalling) in concrete. The method consists of analyzing the interaction between a low-frequency pump wave and a high-frequency probe wave, both travelling along the width of the block in opposite directions. This interaction produces a measurable time-of-flight shift in the probe wave, which accumulates along the sampling path. A nonlinear parameter (NL), defined as the spatial gradient of the normalized time shift, is introduced to suppress cumulative propagation effects and improve damage localization. The proposed technique is tested to detect and locate a thermally damaged concrete prism (0.25 × 0.08 × 0.15 m^3^) cast inside a sound concrete block (0.40 × 0.40 × 0.70 m^3^). The results enabled the detection, localization, and characterization of the damaged concrete prism with a sensitivity beyond that of conventional linear ultrasound. The effectiveness of the method is therefore validated, and it demonstrates its potential for damage monitoring of structures in civil and nuclear engineering applications.

## 1. Introduction

Nonlinear acoustics (NLA) is a branch of acoustics that describes nonlinear phenomena in materials. In heterogeneous solids such as concrete, nonlinear behaviour may arise from both interatomic bonds (classical nonlinearity) and imperfect contacts, such as microcracks (non-classical nonlinearity). The nonlinear wave mixing approach investigated in this work primarily exploits these non-classical nonlinear mechanisms, which are highly sensitive to the presence of damage [1,2,3,4].

Techniques based on nonlinear acoustics have many useful applications for monitoring the structural condition of materials in a non-destructive (NDT) manner. Materials such as concrete are inherently nonlinear due to their heterogeneous nature; nonlinearities increase significantly in the presence of defects such as microcracks and macrocracks caused by thermal damage [5,6,7,8,9] or alkali-silica reaction (ASR) [8,10,11].

Nonlinear techniques that mix ultrasonic waves have already proven to be effective in detecting internal defects in concrete [12,13,14]. In this context, the work of Kim et al. [15] highlights the relevance of nonlinear techniques for damage detection in concrete. The study applies a method based on the interaction of two shear waves within a concrete block. When the waves intersect at a specific angle, the presence of microcracks induces a nonlinear interaction that generates an additional longitudinal wave. The amplitude of this wave is proportional to the nonlinearity in the material, particularly in areas affected by alkali-silica reaction (ASR) or thermal degradation. Although this approach provides localized information at the wave interaction point, it requires a precise geometrical configuration of two intersecting shear waves, which may limit its practical implementation on large concrete structures. Another example is the work of Ouvrier-Buffet et al. [16]. Here, the authors detect the presence of a sandstone sphere (8 cm in diameter) in a concrete block measuring 0.40 × 0.40 × 0.70 m^3^ using a nonlinear acoustic method based on the interaction between the probe wave and the pump wave, where the nonlinearity parameter studied is the relative time-of-flight variations (Δ*TOF*/*TOF*_0_). Unlike the present study, their methodology employed a non-collinear pump–probe configuration requiring access to three faces of the concrete block. The trigger delay was adjusted to ensure that the pump wave excited the damaged area before the passage of the probe wave. Their results showed a marked local increase around 8 times more pronounced in the area containing the sphere, revealing locally accentuated nonlinear behavior. However, the measured Δ*TOF*/*TOF*_0_ represents the cumulative nonlinear response along the probe wave propagation path and therefore does not directly provide the depth position of the damaged region. In the present work, this limitation is addressed by introducing a nonlinear parameter (NL) based on the spatial gradient of the normalized time shift, which suppresses cumulative propagation effects and enables in-depth localization of the nonlinear response.

In the context of the current French nuclear power plant operating life extension, the French Authority for Nuclear Safety and Radiation Protection (ASNR) currently runs several research programs dedicated to concrete and its safety function. In particular, regarding concrete aging, the ODOBA project [17] focuses on swelling reactions (ISR) and their consequences for massive nuclear structures. This project deals with phenomena involved in the appearance and development of pathologies (ASR, delayed ettringite formation—DEF) and with Non-Destructive Technologies for the detection and monitoring of concrete defects.

This NDT incentive-based research is led in partnership with the Laboratory of Mechanics and Acoustics (LMA) and the MISTRAS Group. It aims to develop innovative Non-Destructive Technology for massive structures. The objective of this study is to highlight a possible solution for assessing the ageing of nuclear concrete structures and to expand its scope of applications in civil engineering Structural Health Monitoring (SHM).

Based on the considerations presented above, this study is intended as a proof of concept and represents an intermediate stage of technological development (TRL 4) [18].

The main objective of this work is to develop and validate a nonlinear-acoustics-based non-destructive testing method capable of detecting and locating internal defects within a massive concrete block, thereby contributing to the development of diagnostic and predictive tools for damage evolution in nuclear-grade concrete structures.

In this study, a nonlinear wave mixing approach is proposed for the in-depth localization of internal damage in concrete. The obtained results are in good agreement with previously reported studies [16,19] and overcome some of their limitations, confirming the potential of the proposed methodology.

## 2. Rationale for the Proposed Approach for Internal Damage Localization in Concrete

The proposed method is based on the interaction of two counter-propagating waves. A high-frequency (probe) wave is followed by the interference of a low-frequency (pump) wave, both operating collinearly but in opposite directions (only accessing two sides of the concrete block).

By means of this interaction, it is possible to measure the time-of-flight shift in the probe wave induced by the pump wave, which perturbs the elastic properties of the medium and delays the propagation of the probe wave in the presence of damage. Since pulse wave velocity (Vp) and time-of-flight (*TOF*) are inversely related ($V_{p}=L/TOF$), where L is the constant propagation distance, the relative velocity variation can be expressed in terms of the relative time-of-flight shift, as shown in Equation (1).

(1) $$\frac{\Delta Vp}{Vp}=-\frac{\Delta TOF}{TOF_{0}}$$

This relation is commonly used in nonlinear ultrasonic studies to convert time-of-flight variations into relative velocity changes, which are directly related to nonlinear elastic behavior [20,21].

Changing the interaction position of the pump and probe waves is proposed in this study to detect and locate internal defects within a concrete block. When the two waves interact, the low-frequency pump wave induces a temporary perturbation of the elastic properties of the medium, which modifies the propagation velocity of the high-frequency probe wave. This interaction is expected to produce a measurable variation in the probe wave time-of-flight. The resulting time shift reflects the nonlinear elastic response of the material and can therefore be used as an indicator of structural integrity. This means that by controlling the interaction depth and analyzing the spatial variation in the time-of-flight deviation, the proposed approach should enable the localization of nonlinear sources associated with damaged regions inside the material.

## 3. Materials and Methods

### 3.1. Concrete Blocks Characteristics

**Figure 1:**
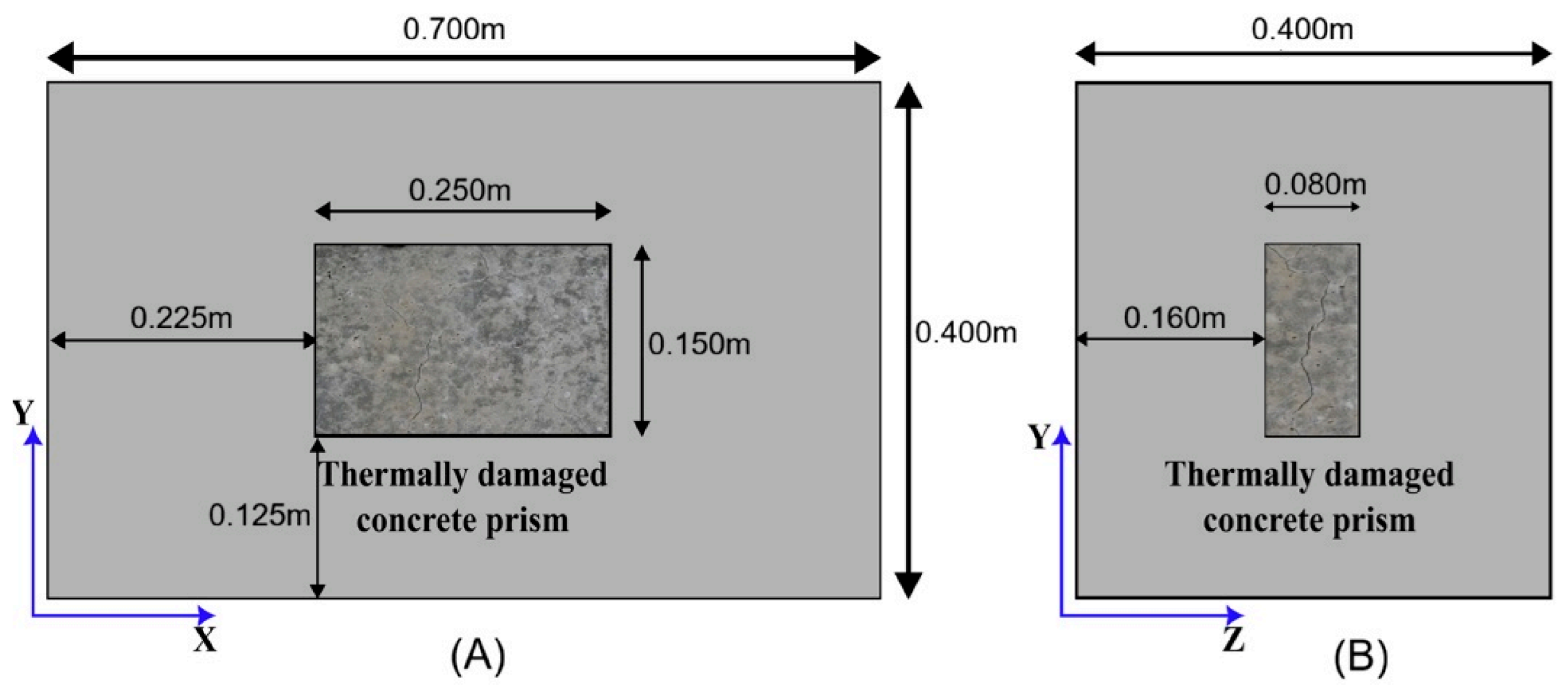
Diagram of a sound concrete block containing a thermally damaged concrete prism: (**A**) front view and (**B**) side view.

For the development of the methodology outlined in this study, a concrete block weighing around 250 kg and measuring 0.40 × 0.40 × 0.70 m^3^ was specifically chosen. Within this block, there is a 0.25 × 0.08 × 0.15 m^3^ thermally damaged (severe cracking) concrete prism, as illustrated in Figure 1. This thermally damaged concrete prism was produced by subjecting the specimen to a controlled heating cycle up to 400 °C in a laboratory furnace. The temperature was increased at 1 °C/min until the target temperature was reached, which was maintained for 3 h to ensure uniform heating of the material. The specimen was then cooled down at a similar rate. This thermal treatment induces microcracking and degradation of the cement matrix and the aggregate–paste interface, resulting in a significant increase in material nonlinearity.

The table below (Table 1) presents a comprehensive overview of the properties of the sound concrete block and the thermally damaged concrete prism within the block.

It should be noted that the embedded thermally damaged concrete prism introduces not only a distributed internal crack network but also an interface between the freshly cast concrete and the thermally damaged concrete prism. This interface constitutes an acoustic impedance contrast due to differences in properties (density and wave velocity) between the thermally damaged and the freshly cast concrete and bonding imperfections. Such imperfections cannot be entirely eliminated. In this study, the concrete prism was soaked in water to prevent the thermally damaged prism from absorbing water from the freshly cast concrete, which would affect hydration and consequently bond quality at the interface. Furthermore, the freshly cast concrete was thoroughly vibrated to promote good interfacial bonding.

### 3.2. Experimental Protocol for Collinear Wave Mixing Method

The experimental protocol presented here focuses on exploiting the interaction between two types of longitudinal waves—the probe wave (transient wave) and the pump wave—to determine the location of damage. The probe wave operates at 100 kHz, while the pump frequency was selected as 28 kHz, corresponding to the resonance frequency of the transducer. This frequency also provides the best compromise between a pump wavelength significantly larger than that of the probe wave and sufficiently small with respect to the specimen dimensions to ensure effective wave interaction within the concrete block. The frequency difference facilitates the isolation of the pump and probe waves during signal processing.

**Figure 2:**
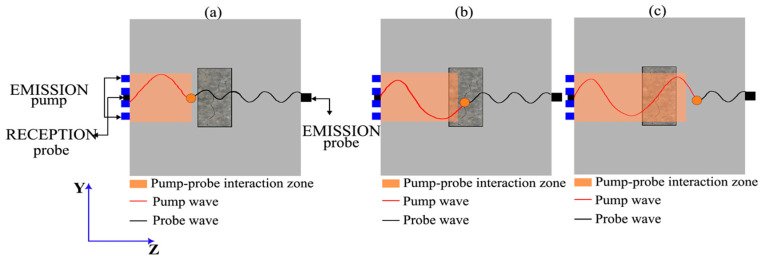
Principle of the experimental protocol showing three examples of pump–probe interaction positions within the block thickness: (**a**) interaction in sound concrete before the damaged prism, (**b**) interaction at the damaged prism depth, and (**c**) interaction beyond the damaged prism. The orange region represents the pump–probe interaction zone.

To investigate the proposed methodology under different propagation conditions, the sample was examined along three measurement paths, each comprising seven measurement positions, allowing different wave propagation paths and interaction depths to be explored. The objective of this experimental protocol is to determine a time shift that accurately reflects the material nonlinear properties, measured over a monitoring interval beginning at the point of wave intersection (orange zone in Figure 2).

By defining specific wave interaction points, the interpretation of the nonlinear characteristics of the material at different depths inside the block can be enhanced, thereby enabling sensitive detection and localization of the defect. In this framework, to control the depth at which the two counter-propagating waves start interacting, a trigger delay $\Delta t_{d}$ was applied between pump and probe emissions. This trigger delay (Equation (4)) considers the P-wave velocity values obtained for the sound concrete and for the thermally damaged concrete prism. Since both pump and probe are longitudinal (P) waves, they are assumed to propagate with the same local P-wave velocity Vp in concrete. The influence of dispersion is expected to be limited and is considered part of the overall uncertainty of the proposed methodology. For a prescribed interaction depth z within a specimen of thickness L, the travel times required for each wave to reach z are:

(2) $$t_{\mathrm{probe}}\left(z\right)=\frac{L-z}{Vp}$$

(3) $$t_{\mathrm{pump}}\left(z\right)=\frac{z}{Vp}$$

To ensure temporal overlap at depth z, the relative trigger delay was set to:

(4) $$\Delta t_{d}=t_{\mathrm{probe}}\left(z\right)-t_{\mathrm{pump}}\left(z\right)$$

The prescribed interaction depth, Z, corresponds to the position at which the pump and probe wave packets begin to overlap. The trigger delay was adjusted to shift this onset position through the specimen thickness in 0.04 m increments. Depending on the prescribed depth, either the pump or the probe emission was delayed so that both wave packets reached the selected location simultaneously. This procedure was repeated for each 0.04 m increment to scan the entire specimen thickness. Since the interaction occurs over a finite spatial region governed by the duration of the wave packets, uncertainties in the local P-wave velocity, which are inherent to ultrasonic wave propagation in heterogeneous concrete and to time-of-flight estimation, propagate into the estimated interaction depth and may slightly shift or broaden the effective interaction zone. A recent work [22] has highlighted the importance of accounting for these uncertainty sources in ultrasonic wave-speed measurements in concrete. In addition, uncertainties in the trigger-delay adjustment contribute to the localization uncertainty. Nevertheless, these effects remain limited and do not compromise the ability of the proposed methodology to localize the damaged region.

Diagrams a, b, and c in Figure 2 show the underlying principles of the experimental protocol, each corresponding to a distinct case in which the waves intersect at different depths. The experimental tests were performed at 0.04 m thickness intervals, thus sweeping all areas of the block, both sound and damaged regions, to assess the material properties at multiple depths.

Given the inherently nonlinear behavior of concrete, a cumulative time-shift effect is expected; specifically, the longer the wave interaction duration, the greater the shift induced by the intrinsic nonlinearity of the material. In addition, with respect to the thermally damaged concrete prism, the anticipated effect is that when the waves intersect within or beyond it, a pronounced increase in the time shift occurs, attributable to the contrasting nonlinearity.

### 3.3. Experimental Setup

#### 3.3.1. Instrumentation

**Figure 3:**
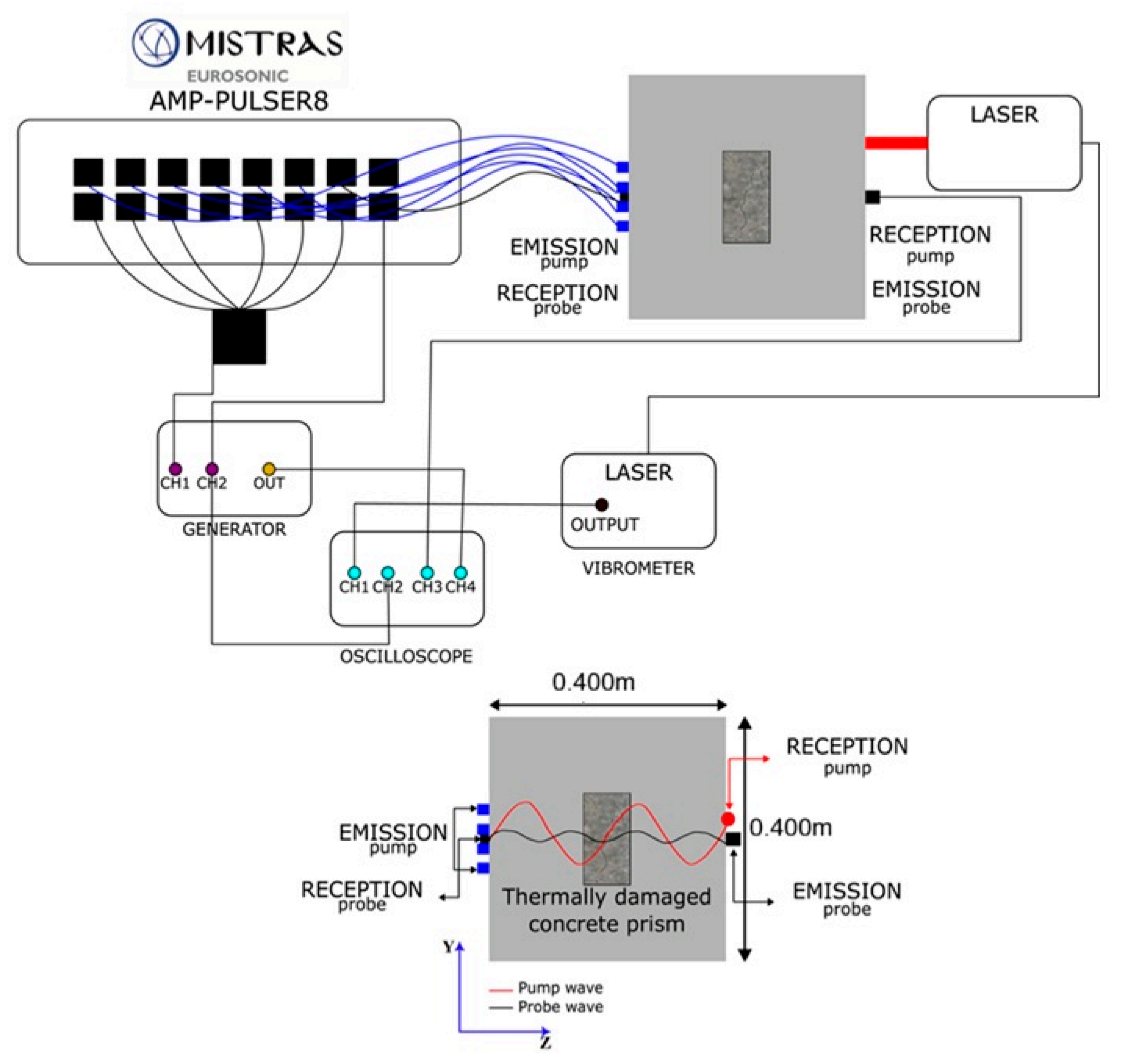
Instrumentation diagram for a concrete block.

**Figure 4:**
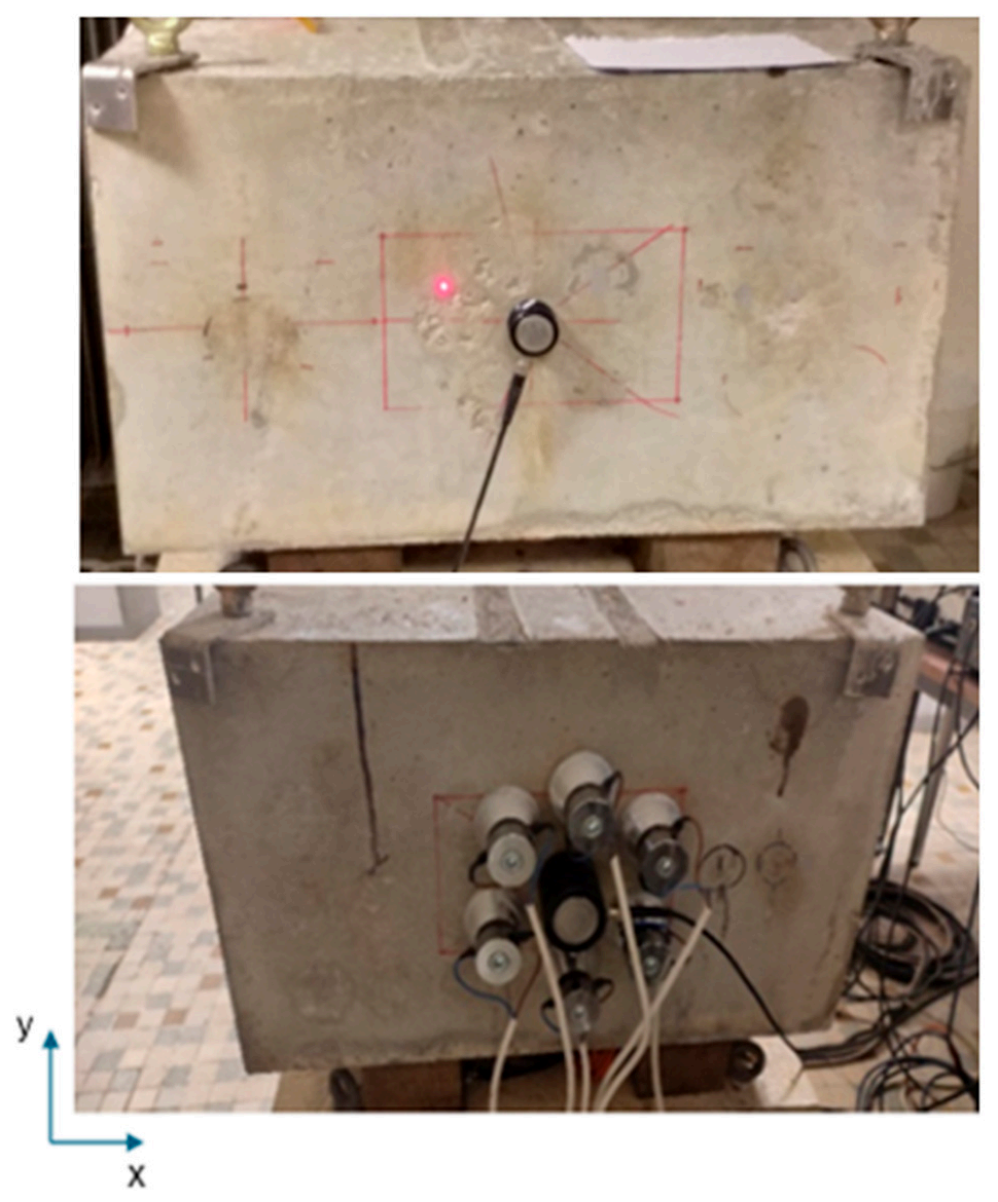
Experimental setup showing the circular array of 6 pump transducers mounted on one face of the concrete block. The central transducer is used to receive the probe wave, while the probe is emitted from the opposite face of the concrete block, where there is only one transducer.

Figure 3 shows a left view of the block, delineating the instrumentation system designed for both pump and probe waves. In addition, Figure 4 provides a front- view representation of the block at a designated measurement region corresponding to the zone containing the thermally damaged concrete prism. As seen in the figure, the pump wave was generated using a circular array of six contact piezoelectric transducers (cleaner-type) with a nominal resonant frequency of 28 kHz. The probe wave was generated using a Panametrics V1011 contact transducer (Olympus IMS, Waltham, MA, USA) with a nominal resonant frequency of 100 kHz and a diameter of 38.1 mm (1.5 inches). Both types of transducers were coupled to the concrete surface using phenyl salicylate, following a coupling procedure similar to that adopted in Ouvrier-Buffet et al. [16,19]. The transducers were maintained in position under controlled contact conditions during the measurements.

For signal generation, an AFG-3102 dual-channel function generator (Tektronix Inc., Beaverton, OR, USA) was utilized to produce both the pump and probe waves. The instrument enables fine adjustment of the trigger delay to ensure accurate temporal synchronization and interaction between the two signals. The pump wave was received using a Polytec OFV-505 laser vibrometer (Polytec GmbH, Waldbronn, Germany), which measures particle velocity along the beam axis with a sensitivity of 25 mm·V^−1^·s^−1^. For signal acquisition and data recording, all instruments were synchronized via a Lecroy Teledyne HDO 4024 oscilloscope (Teledyne LeCroy, Chestnut Ridge, NY, USA) with a sampling rate of 10 GS/s, which was largely sufficient for the measured TOF and ΔTOF values.

To optimize measurements and increase the power of the system, an amplifier called AMP-PULSER8© (MISTRAS Group SAS, Sucy-en-Brie, France) was designed to integrate the acquisition setup used for this method. This device has a significant advantage: it can amplify low-frequency channels separately. In the case at hand, it simultaneously amplifies both types of waves on the same device. The six pump wave transducers are connected to six independent channels, as is the probe via an independent channel. This allows the emitted signal to be amplified up to 85 times, making the system particularly powerful for large thicknesses.

#### 3.3.2. Acquisition of Time Shift Data

The data acquisition process is based on Gallot et al. [23], and the sequence unfolds as follows:

I. Initially, the probe wave is activated, emitting 03 sinusoidal cycles at 100 kHz, which travel across the material without any interference from the pump wave;
II. Subsequently, the pump wave propagates through the material for 05 sinusoidal cycles at 28 kHz without activating the probe wave. This deliberate separation allows the pump-wave contribution to the received signal to be identified and removed during post-processing, ensuring that only the effect of the pump–probe interaction is analyzed;
III. Finally, the probe wave is transmitted through the material while the pump wave is concurrently activated, resulting in pump-probe wave interaction. This interaction is particularly accentuated in regions characterized by significant nonlinearities, such as the cracks and micro-cracks present in the embedded thermally damaged concrete prism.

The reference time-of-flight (*TOF*_0_) was determined from the probe signal acquired without pump excitation by identifying its arrival time through the maximum of the cross-correlation with the emitted probe waveform. Each perturbed probe signal was subsequently cross-correlated with this reference signal to determine the relative time shift Δ*TOF*. The results for the relative time-of-flight (Δ*TOF*/*TOF*_0_) are the average of three independent measurements taken at each wave interaction point within the block thickness, while the error bars represent one standard deviation of these measurements.

## 4. Results and Discussion

### 4.1. Initial Assessment

**Figure 5:**
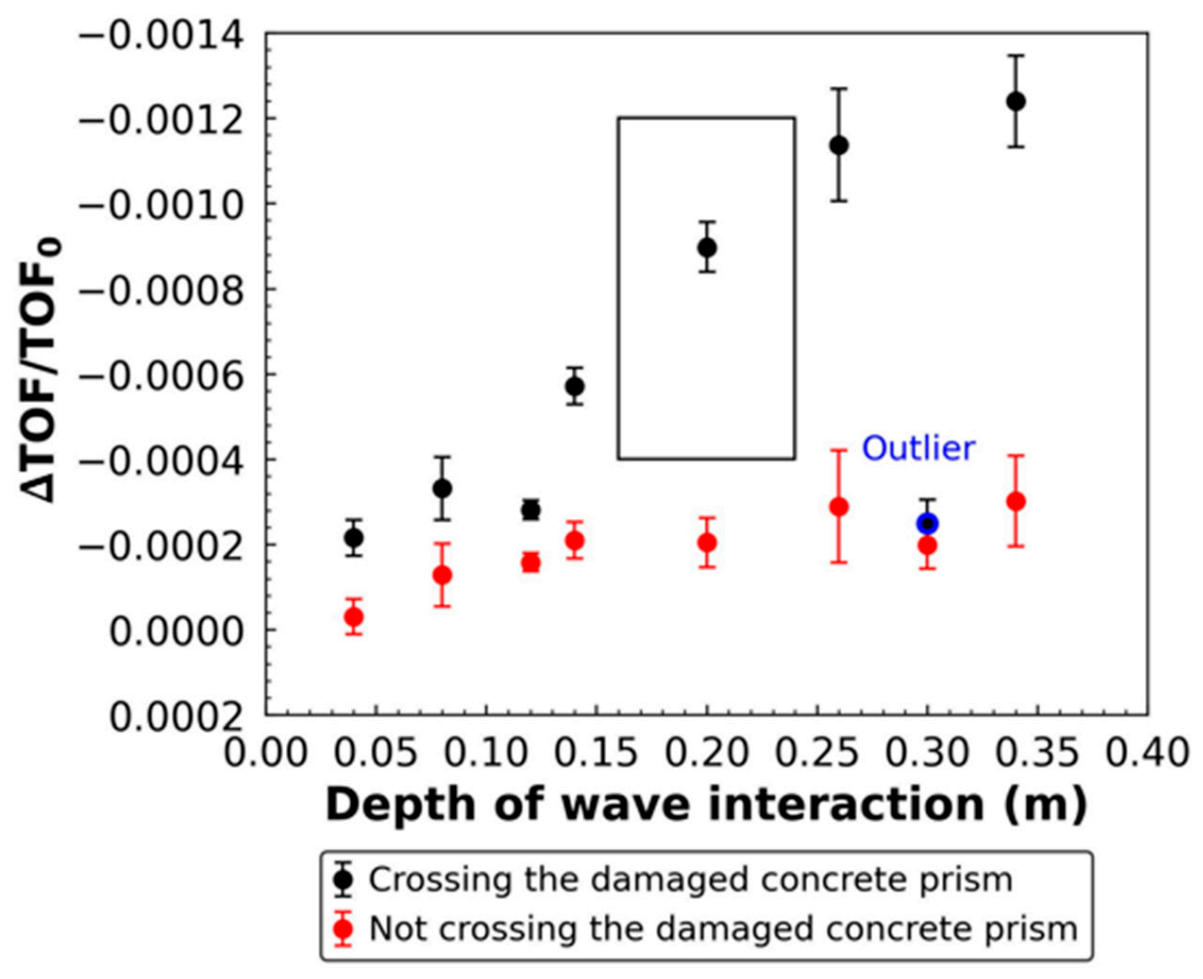
Relative time shift as a function of the wave interaction position in sound and damaged concrete zones.

The graph in Figure 5 shows the relative time-of-flight results for waves traveling across the sound zone of the block (red dots) and going through the area containing the thermally damaged concrete prism (black dots).

The points observed in the graph correspond to each interaction point. The results of the time-of-flight shift are an average of the local mechanical integrity of the block in the zone between the interaction point and the signal reception. Error bars represent the standard deviation of the three replicate measurements for the Δ*TOF*/*TOF*_0_ measurements.

Regarding the thickness of the entire interaction zone starting from the interaction point, the point located at X = 0.04 m, for example, is located at this distance from the probe receiving face. Hence, both pump and probe waves interact over this 0.04 m. The same applies to all points represented below.

The results show that at a depth of 0.2 m, corresponding to the center of the block and the thermally damaged zone, there is a clear difference between the |Δ*TOF*/*TOF*_0_| values measured in the sound concrete (red points) and in the thermally damaged concrete (black points) zones. As shown in Figure 5, the relative time-of-flight shift |Δ*TOF*/*TOF*_0_| reaches approximately 9 × 10^−4^ in the thermally damaged concrete zone, while it remains around 2 × 10^−4^ in the sound concrete. This indicates that the nonlinear response in the damaged zone based on the relative time-of-flight shift is about 4.4 times higher than in the undamaged zone, highlighting a strong amplification associated with the presence of microcracks and macrocracks within the thermally damaged concrete prism.

This enhanced response is induced by the low-frequency pump wave, which perturbs the elastic properties of the material and increases the sensitivity of the probe wave to local material nonlinearity. However, the measured |Δ*TOF*/*TOF*_0_| remains a cumulative parameter, as the nonlinear interaction accumulates along the propagation path. Consequently, |Δ*TOF*/*TOF*_0_| alone cannot directly localize the damaged region.

These results show that the nonlinear method is more sensitive than linear acoustics and that nonlinear effects accumulate over propagation time. This cumulative effect is observed even when the waves interact only within the sound concrete part of the block, as it depends on the amount of material involved in the wave interaction and therefore increases with the depth of interaction. This is clearly illustrated by the progressive increase in both |Δ*TOF*/*TOF*_0_| curves over depth in Figure 5.

For the region of the block not containing the thermally damaged concrete prism (red points), the difference between the initial (0.04 m) and final (0.34 m) |Δ*TOF*/*TOF*_0_| values corresponds directly to the aforementioned cumulative effect. The cumulative effect is 2.7 × 10^−4^, and the increase remains relatively linear. For the region of the block containing the thermally damaged concrete prism (black points), this value reaches 1.0 × 10^−3^, which corresponds to a cumulative effect almost ×4 greater than the one obtained in the region of the block not containing the thermally damaged concrete prism. In addition, the increase is no longer linear and shows a sharp inflection near the thermally damaged block.

### 4.2. Definition of a Local Indicator of Nonlinearity, the Nonlinear Parameter NL

However, the relative time-of-flight shift obtained from the pump-probe interaction presented in Section 4.1 remains a cumulative quantity along the sampling paths. To remove this effect and obtain a local indicator of nonlinearity, a nonlinear parameter, NL, is proposed.

This parameter is defined as the spatial derivative of the relative time-of-flight variation over the position of the interaction (dz), as shown in Equation (5).

(5) $$NL=-\frac{d\left(\frac{\Delta TOF}{TOF_{0}}\right)}{dz}$$

**Figure 6:**
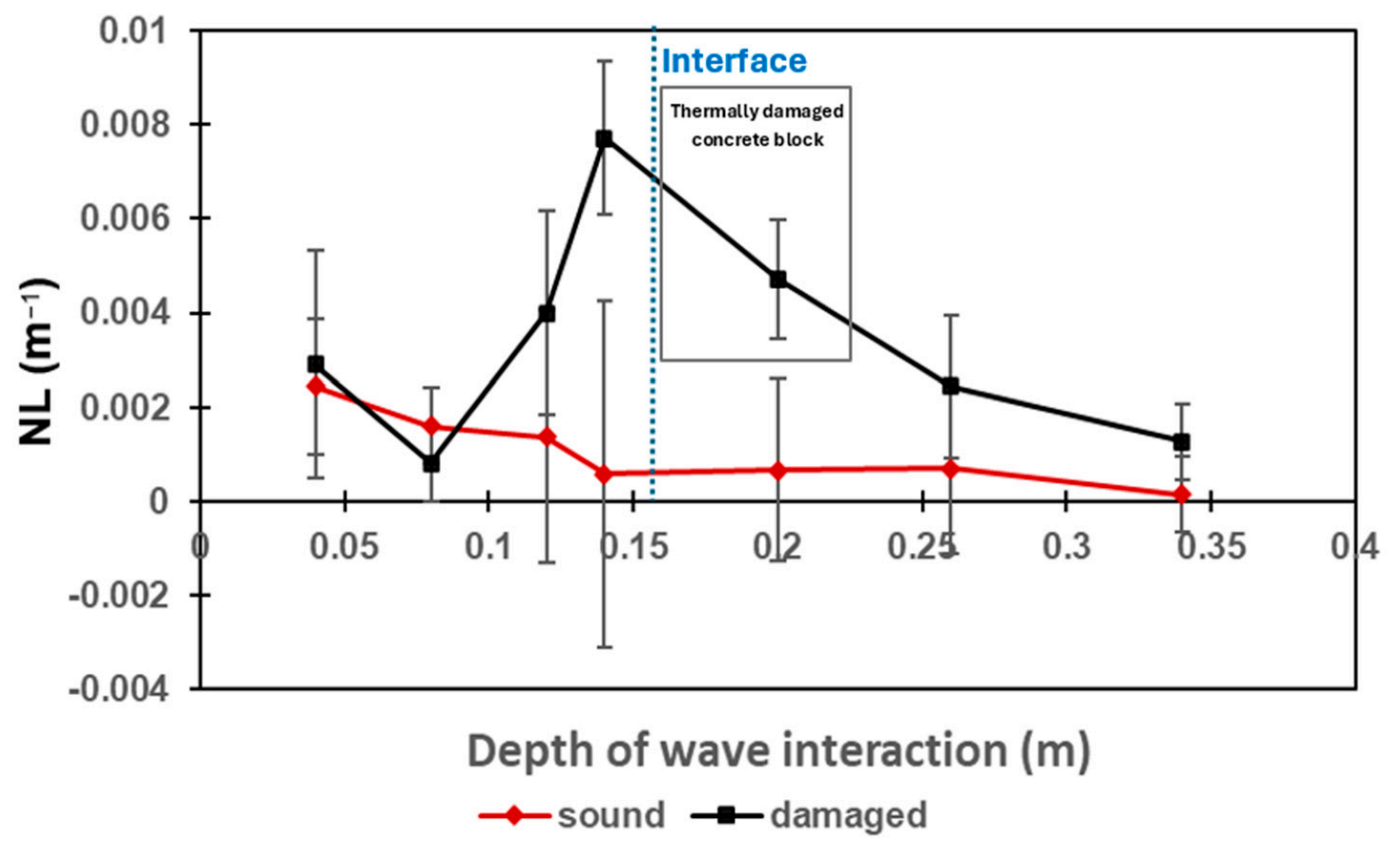
Depth profile of the nonlinear parameter NL as a function of the pump–probe interaction depth.

Figure 6 shows the results for the proposed NL parameter. The derivative of the relative time-of-flight shift with respect to depth was computed using a central finite difference for interior points and forward/backward differences at the boundaries to eliminate the cumulative effect (see Figure 5 and Section 4.1) and better locate the damaged block. The NL parameter was calculated independently for each of the three repeated acquisitions performed at every interaction depth. The values reported in Figure 6 correspond to the mean of these three independent calculations, while the error bars represent the standard deviation (SD).

Since NL is obtained from the numerical differentiation of the measured normalized time-of-flight variation, it is inherently more sensitive to experimental noise than the original quantity. Nevertheless, despite this increased variability, the damaged region remains clearly distinguishable from the sound region.

The black curve shown in Figure 6 clearly reveals a localized increase in nonlinearity in measurements where waves interact near the interface, corresponding to the thermally damaged concrete prism location. The red curve exhibits a quasi-constant profile with NL values comparable to those obtained in the black curve when the interaction occurs outside of the thermally damaged concrete prism. The slight decrease in the NL parameter with depth can be attributed to attenuation, which is not accounted for in the present study. For this block, the attenuation coefficient was estimated, in a previous study, by Ouvrier-Buffet [19]. This coefficient is approximately 1.1 Np/m for the pump wave propagation along the measurement direction (i.e., through the specimen thickness). Over the propagation distance considered in the present study (0.4 m). This level of attenuation remains moderate, and the acoustic power of the excitation system remains sufficient to generate a detectable nonlinear interaction.

**Figure 7:**
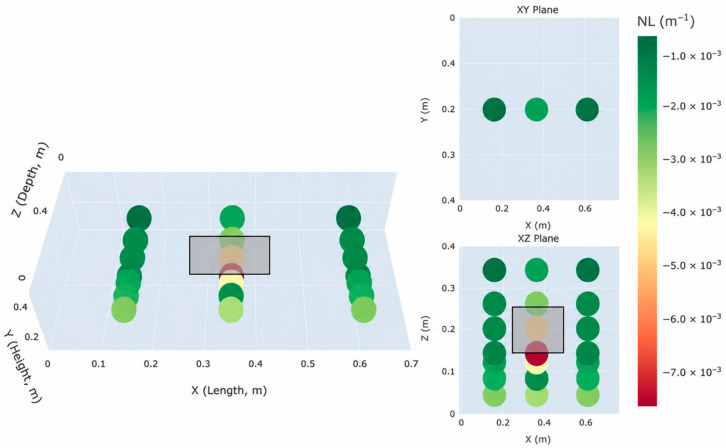
Spatial visualization of the NL parameter.

Figure 7 shows the same NL parameter plotted against real depth (in meters). This representation keeps the physical dimensions and directly locates the damaged area between 16 cm and 24 cm in the 40 cm-thick sample. A slight increase in the derivative is observed near the thermally damaged concrete prism interface (around 12–15 cm). This effect is attributed to wave–interface interaction, which can promote nonlinear effects. It remains consistent with the depth localization. This behavior is consistent with the strong mechanical contrast and the presence of microcracks at the damaged interface, which increases the nonlinear interaction between the pump and probe waves. Despite this slight shift, the detected high-nonlinearity zone remains in good agreement with the actual location of the thermally damaged concrete prism.

Each point corresponds to the position where the waves begin to interact in the YZ plane, and the value obtained corresponds to the derivative of the time-of-flight shift average presented above in Figure 5.

A notable concentration of high NL values (yellow, orange, and red) can be observed around the center of the volume, consistent with the location of the thermally damaged concrete prism.

This visualization confirms the presence of a localized thermally damaged block. It highlights the effectiveness of the counter-propagating wave mixing method for detecting and mapping internal defects in depth/thickness in a non-destructive manner. Moreover, the NL parameter allows quantification of the amount of nonlinearity. On average, the NL values measured within the damaged region (interaction depths between 0.14 and 0.26 m) are approximately 5–7 times higher than those measured in the surrounding sound concrete, demonstrating the strong damage-induced contrast provided by the proposed approach.

### 4.3. Qualitative Comparative Assessment of the Proposed Method and Existing Literature

To position the proposed approach relative to existing nonlinear ultrasonic techniques, Table 2 summarizes a qualitative comparison of the proposed method with several reported techniques.

Although nonlinear ultrasonic techniques have demonstrated high sensitivity for detecting microstructural damage in concrete, many existing approaches provide mainly global indicators and do not allow straightforward depth localization of nonlinear sources within thick structural elements. The present study addresses this limitation by introducing a collinear counter-propagating pump–probe wave mixing approach combined with a local nonlinear indicator derived from the spatial variation in relative time-of-flight. This formulation enables the detection and depth localization of damaged regions inside concrete structures while maintaining a relatively simple experimental configuration suitable for structural health monitoring applications.

The experimental results obtained with the proposed approach demonstrate a clear contrast between sound and damaged zones, confirming the capability of the proposed nonlinear indicator to identify internal damage in concrete structures. The method also shows potential for detecting early-stage degradation mechanisms, such as incipient thermal microcracking or alkali–silica reaction (ASR), before large-scale cracking develops on the surface.

It should be noted, however, that the present study was conducted on a single intermediate-scale concrete specimen. Consequently, the repeated measurements reported evaluate the repeatability of the proposed methodology but do not assess specimen-to-specimen variability. Moreover, using multiple propagation paths minimizes the influence of local spatial variability associated with concrete heterogeneity and specimen fabrication, independently of operator effects or differences in concrete formulation between specimens cast at different times. This approach is consistent with the objective of the present work, namely to introduce the proposed collinear pump–probe methodology and demonstrate its capability to localize damaged regions under controlled conditions. The present work constitutes an intermediate step within a broader research program dedicated to the development of nonlinear ultrasonic methods for structural health monitoring of nuclear concrete structures. Future studies will extend the proposed methodology to larger structural-scale specimens and will investigate specimen-to-specimen variability as well as long-term monitoring capabilities.

## 5. Conclusions and Perspectives

This study advances nonlinear acoustic methods for concrete inspection by validating a depth-localization and characterization approach under controlled conditions. The proposed depth localization method based on counter-propagating wave mixing in nonlinear acoustics has demonstrated, at an intermediate laboratory scale, its ability to accurately localize defects within concrete structures. The results show a clear contrast between sound and damaged zones, demonstrating a substantially higher sensitivity than conventional linear ultrasonic measurements.

Beyond these initial results, the method presents a manageable level of experimental complexity and shows promising potential for adaptation to larger concrete structures. As part of the ODOBA project [17], this phase has validated the launch of tests on a 10-ton concrete block with a thickness of approximately 1 m, damaged by alkali–silica reaction (ASR), to approximate real-scale structural conditions. These large-scale experiments will aim to evaluate the capability of the technique to detect and localize degradation mechanisms associated with ASR in thicker concrete elements.

Particular attention will be given to the influence of structural heterogeneity and swelling-induced microcracking on the pump–probe nonlinear interaction. A major challenge for large-scale applications will be related to the maximum acoustic power of the excitation system and to the long-term stability of the coupling conditions under environmental exposure and aging cycles.

In addition, future investigations will aim to better characterize the sensitivity threshold of the NL parameter for early-stage damage and to assess the robustness of the method under conditions representative of in-situ structural health monitoring.

In the long term, this approach offers valuable prospects for detecting real structural degradation associated with material pathologies—such as internal swelling reactions—rather than solely thermally induced effects simulated under controlled laboratory conditions.

Overall, the results highlight the potential of nonlinear acoustic approaches for advanced structural health monitoring. Although the method provides enhanced sensitivity and more accurate damage localization compared to conventional linear acoustic methods, further investigation of its sensitivity limits is required to evaluate the potential for earlier damage detection. This study represents an important step toward the practical deployment of nonlinear acoustics for large-scale civil engineering structures.

## Figures and Tables

**Table 1:** Undamaged and damaged concrete properties.

Material Properties	Undamaged Concrete	Thermally Damaged Concrete Prism
Density (kg/m^3^)	2400	2050
Poisson’s Ratio	0.25	0.13
Young’s Modulus (GPa)	45.5	9
S-wave velocity (m/s)	2758	1400
P-wave velocity (m/s)	4762	2150

**Table 2:** Qualitative comparison between the proposed nonlinear ultrasonic method and representative nonlinear acoustic techniques reported in the literature, considering the nonlinear indicator used, spatial localization capability, sensitivity, experimental complexity, and potential applicability to in-situ monitoring of concrete structures.

Method	Nonlinear Indicator	Spatial Localization	Sensitivity	Experimental Complexity	In-Situ Applicability
Non-collinear wave mixing (Kim et al., 2022) [15]	Generated nonlinear wave amplitude	Localized interaction zone (2D)	High	High (precise angular alignment required)	Limited (Weak nonlinear amplitude for thick elements)
Non-collinear wave mixing (Kumar et al., 2025) [13]	Generated nonlinear wave amplitude	Localized interaction zone (2D)	High	High (precise angular alignment required)	High (precise angular alignment required)
Nonlinear resonant ultrasound (Yang et al., 2019) [7]	Resonance frequency shift	Global (no depth localization)	Very high	Moderate	Limited (global indicator)
Pump–probe wave mixing (Ouvrier-Buffet et al., 2024) [16]	ΔTOF/TOF_0_ variation	Localized nonlinear zone (2D)	High	Moderate–high	difficult to apply on site. Requires access to multiple faces
Proposed method	NL = −d(ΔTOF/TOF_0_)/dz	Depth localization	High	Moderate (requires signal processing)	Promising for large concrete structures and early detection

## Data Availability

The data presented in this study are available from the corresponding author upon reasonable request.
